# Observation of topologically protected states at crystalline phase boundaries in single-layer WSe_2_

**DOI:** 10.1038/s41467-018-05672-w

**Published:** 2018-08-24

**Authors:** Miguel M. Ugeda, Artem Pulkin, Shujie Tang, Hyejin Ryu, Quansheng Wu, Yi Zhang, Dillon Wong, Zahra Pedramrazi, Ana Martín-Recio, Yi Chen, Feng Wang, Zhi-Xun Shen, Sung-Kwan Mo, Oleg V. Yazyev, Michael F. Crommie

**Affiliations:** 1 Donostia International Physics Center (DIPC), Manuel Lardizábal 4, 20018 San Sebastián, Spain; 2 Centro de Física de Materiales (CSIC-UPV/EHU), Manuel Lardizábal 5, 20018 San Sebastián, Spain; 3 Ikerbasque, Basque Foundation for Science, 48013 Bilbao, Spain; 4 Institute of Physics, Ecole Polytechnique Fédérale de Lausanne (EPFL), CH-1015 Lausanne, Switzerland; 5 Advanced Light Source, Lawrence Berkeley National Laboratory, Berkeley, CA 94720 USA; 6 Stanford Institute for Materials and Energy Sciences, SLAC National Accelerator Laboratory, Menlo Park, CA 94025 USA; 7 Center for Spintronics, Korea Institute of Science and Technology, Seoul, 02792 Korea; 8 National Centre for Computational Design and Discovery of Novel Materials MARVEL, Ecole Polytechnique Fédérale de Lausanne (EPFL), CH-1015 Lausanne, Switzerland; 9 National Laboratory of Solid State Microstructures, School of Physics, Collaborative Innovation Center of Advanced Microstructures, Nanjing University, Nanjing, 210093 China; 10 Department of Physics, University of California at Berkeley, Berkeley, CA 94720 USA; 11 Departamento de Física de la Materia Condensada, Universidad Autónoma de Madrid, E-28049 Madrid, Spain; 12 Materials Sciences Division, Lawrence Berkeley National Laboratory, Berkeley, CA 94720 USA; 13 Kavli Energy NanoScience Institute at the University of California Berkeley and the Lawrence Berkeley National Laboratory, Berkeley, CA 94720 USA; 14 Geballe Laboratory for Advanced Materials, Departments of Physics and Applied Physics, Stanford University, Stanford, CA 94305 USA

## Abstract

Transition metal dichalcogenide materials are unique in the wide variety of structural and electronic phases they exhibit in the two-dimensional limit. Here we show how such polymorphic flexibility can be used to achieve topological states at highly ordered phase boundaries in a new quantum spin Hall insulator (QSHI), 1*T*′-WSe_2_. We observe edge states at the crystallographically aligned interface between a quantum spin Hall insulating domain of 1*T*′-WSe_2_ and a semiconducting domain of 1*H*-WSe_2_ in contiguous single layers. The QSHI nature of single-layer 1*T*′-WSe_2_ is verified using angle-resolved photoemission spectroscopy to determine band inversion around a 120 meV energy gap, as well as scanning tunneling spectroscopy to directly image edge-state formation. Using this edge-state geometry we confirm the predicted penetration depth of one-dimensional interface states into the two-dimensional bulk of a QSHI for a well-specified crystallographic direction. These interfaces create opportunities for testing predictions of the microscopic behavior of topologically protected boundary states.

## Abstract (web summary)

Transition metal dichalcogenides may host exotic topological phases in the two-dimensional limit, but detailed atomic properties have rarely been explored. Here, Ugeda et al. observe edge-states at the interface between a single layer quantum spin Hall insulator 1*T*′-WSe_2_ and a semiconductor 1*H*-WSe_2_.

## Introduction

Materials exhibiting the quantum spin Hall effect (QSHE) create new opportunities for directly imaging the spatial extent of topologically protected one-dimensional (1D) edge states and for determining how they interact with bulk states and defects. Such systems, however, can be difficult to isolate and to access via microscopy. HgTe and InAs/GaAs quantum wells, for example, are well-known QSHIs^1,2^, but are not easily accessible to high-resolution scanned probe microscopy because they are buried interface systems. Bi-based surface systems (predicted to be QSHIs^3,4^) have shown evidence for QSHI behavior and are more accessible to scanned probe microscopy, but exhibit strong substrate interactions^5–7^. Monolayer transition metal dichalcogenide (TMD) materials (*MX*_2_ where *M* = Mo, W, and *X* = S, Se, and Te) in the distorted octahedral 1*T*′ phase, on the other hand, are a new class of QSHIs^8^ that retain their topological properties on different substrates and are completely accessible to high-resolution scanned probe microscopy^9–11^. Monolayer 1*T*′-WTe_2_ films have recently been shown to exhibit all of the hallmarks of the QSH effect (e.g., band inversion, helical edge states, and edge-state quantum conduction) via angle-resolved photoemission spectroscopy (ARPES)^9^, scanning tunneling microscopy/spectroscopy (STM/STS)^9–11^, and transport measurements^12,13^. Monolayer 1*T*′-WTe_2_, however, poses challenges for quantitative microscopy of topological edge states due to the high degree of structural disorder in the edges of 2D 1*T*′-WTe_2_ islands produced by molecular beam epitaxy (MBE). Although the existence of topological edge states is protected against disorder, quantitative characterization of their decay lengths, dispersion features, and defect interactions requires crystallographically well-ordered edges since these properties strongly depend on edge orientation^14–16^, strain, and chemical environment^17^.

**Fig. 1:**
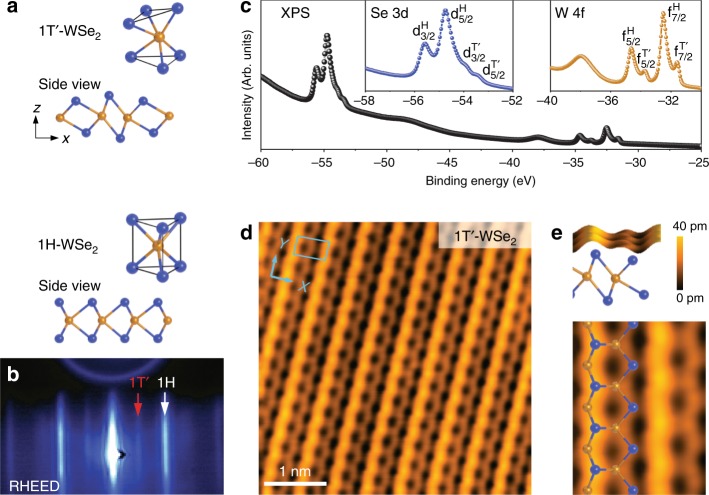
Atomic structure of mixed-phase single-layer WSe_2_. **a** Calculated unit cells and side-view sketches of the 1*T*′ and 1*H* phases of single-layer WSe_2_. Se (W) atoms are depicted in blue (orange). **b** RHEED pattern of single-layer 1*T*′/1*H*-mixed-phase WSe_2_. Red and white arrows indicate diffraction stripes from 1*T*′ and 1*H* phases, respectively. **c** Core-level XPS spectrum of single-layer 1*T*′/1*H* mixed-phase WSe_2_. Insets show zoom-in of the Se (blue) and W (orange) peaks for the 1*T*′ (*d*^*T’*^, *f*^*T’*^) and 1*H* (*d*^*H*^, *f*^*H*^) phases. **d** Atomically resolved STM image of single-layer 1*T*′-WSe_2_. The unit cell is indicated in blue (*V*_s_ = +500 mV, *I*_t_ = 1 nA). **e** Side and top view close-up of the 1*T*′-WSe_2_ STM image with a sketch of calculated 1*T*′-WSe_2_ (only upper-layer Se atoms are depicted in top view)

In order to achieve structurally well-defined boundaries in a fully accessible QSHI, we grew mixed-phase WSe_2_ monolayers on SiC(0001) using MBE growth techniques. Single-layer WSe_2_ is bimorphic with two stable crystalline phases (1*H* and 1*T*′ (Fig. 1a)) that are close in energy^8^, thus enabling the growth of mixed topological/trivial phases with crystallographically defined phase boundary interfaces. The 1*H* phase (which is the structural ground state of WSe_2_) has a much larger electronic bandgap^18,19^ than the 1*T*′ phase, thus allowing the two phases to be easily distinguished. The onset of the QSHE in mixed-phase WSe_2_ thus results in topologically protected states at crystallographically well-defined 1*T*′–1*H* phase boundary interfaces. We have verified the QSHI ground state of 1*T*′-WSe_2_ using ARPES, STM/STS, and first-principles calculations. ARPES reveals the existence of inverted bands at the Fermi energy (*E*_F_) and the presence of a bulk bandgap. STS measurements confirm the bulk bandgap seen by ARPES and further demonstrate the existence of topological interface states within this bandgap that are spatially localized at 1*T*′-WSe_2_ boundaries. These boundary states are easily observable at crystallographically well-ordered 1*T*′–1*H* interfaces, but can also be seen at the irregular 1*T*′ edges. The structural perfection of the 1*T*′–1*H* boundary allows us to measure an interface state decay length of 2 nm into bulk 1*T*′-WSe_2_, agreeing with the results of ab initio numerical simulations.

## Results

### Structural characterization of single-layer 1*T*′-WSe_2_

Our experiments were carried out on high-quality single layers of WSe_2_ grown on epitaxial bilayer graphene (BLG) on 6H-SiC(0001) by MBE. In order to obtain the metastable 1*T*′-WSe_2_ phase, the temperature of the BLG/SiC(0001) substrate was held at 500 K during growth, a significantly lower temperature than required to grow the more stable 1*H* phase (675 K). Under these growth conditions the RHEED pattern of single-layer WSe_2_ (Fig. 1b) shows the formation of an additional large lattice periodicity (5.8 Å) consistent with the 1*T*′ phase that coexists with the shorter 1*H* phase periodicity (3.3 Å). XPS measurements of the WSe_2_ layers (Fig. 1c) reveal the emergence of two new pairs of peaks (*d*^*T*′^ and *f*^*T*′^) near the characteristic Se (*d*^*H*^) and W (*f*^*H*^) peaks for the 1*H* phase^19^, suggesting the presence of an additional lattice symmetry for W and Se^20^. STM imaging confirms that our WSe_2_ layers are composed of coexisting domains of 1*H* and 1*T*′ phase (Supplementary Fig. 1). Figure 1d shows an atomically resolved STM image of the 1*T*′ phase of WSe_2_, which is characterized by straight atomic rows of two non-equivalent zigzag atomic chains. The 1*T*′ phase of Fig. 1d exhibits a period enlargement to 5.73 ± 0.09 Å along the *x* direction compared to the 1*H* phase, in good agreement with the RHEED spectra. Adjacent atomic rows in 1*T*′-WSe_2_ exhibit a slight translational shift along the *y*-direction due to a shear angle that varies between 2° and 6° depending on the domain, similar to that observed previously for other TMD materials^21,22^. We identify the atomic rows in the STM images of Fig. 1d, e as originating from W-Se zigzag chains (see sketch in Fig. 1e), in good agreement with the expected structural distortion of the 1*T*′ phase^8^. The ball-and-stick model shown in Figs. 1a, e corresponds to our calculated relaxed atomic structure of 1*T*′-WSe_2_.

### Electronic characterization of single-layer 1*T*′-WSe_2_

**Fig. 2:**
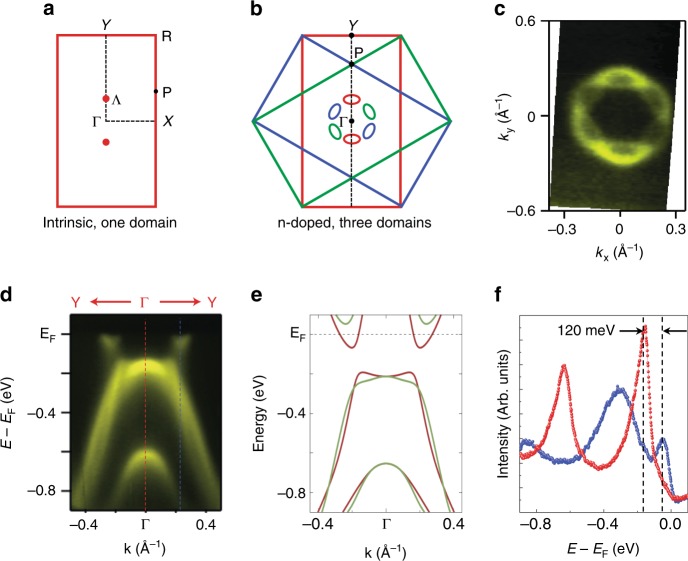
ARPES characterization of single-layer 1*T*′-WSe_2_. **a** Sketch of the first Brillouin zone of 1*T*′-WSe_2_. Relevant high-symmetry points are indicated. **b** Three surface Brillouin zones corresponding to the three rotational 1*T*′-WSe_2_ domains on the BLG surface represented by three different colors. The Fermi surface pockets from each rotational domain are indicated by ellipses of corresponding colors. Black dashed line represents the experimental ARPES line cut shown in **d**. **c** Experimental 1*T*′-WSe_2_ Fermi surface measured by ARPES. **d** High-resolution ARPES band dispersion along the *Y-Γ-Y* direction. Due to the presence of rotational domains, contributions from both *Γ-Y* and *Γ-P* directions are observed in a single ARPES measurement (*T* = 60 K and photon energy *E* = 75 eV). **e** Calculated bands for the 1*T*′ phase of single-layer WSe_2_ along *Γ-Y* (brown) and *Γ-P* (green) directions. A downward rigid shift of 130 meV has been added to account for n-doping seen in the experiment. **f** EDCs from the momentum positions marked with dashed blue and red lines in **d**

We experimentally characterized the electronic structure of coexisting 1*H* and 1*T*′ phases of single-layer WSe_2_ via ARPES and STS. Figure 2c shows the Fermi surface (FS) intensity map for a 0.8 monolayer (ML) coverage of mixed-phase WSe_2_ measured via ARPES. The observed FS structure is entirely due to the 1*T*′ phase since the valence band (VB) maximum of 1*H*-WSe_2_ has a much higher binding energy at *E* = −1.1 eV^19^. The FS is composed of two small elliptical electron pockets at the Λ points located along *ΓY* (Fig. 2a). The three equivalent rotational domains of the 1*T*′ phase on BLG leads to the emergence of three pairs of these features rotated by 120° (Fig. 2b), thus forming a ring-like FS around the *Γ* point. Figure 2d shows the measured band dispersion along the *ΓY* direction of the Brillouin zone (BZ). Due to the rotational domains, contributions from both the *ΓY* and *ΓP* directions can be resolved. The VB maximum is approximately 170 ± 20 meV below the *E*_F_ and exhibits a flattened, non-parabolic onset shape along *ΓY*. Naturally occurring n-type doping in our samples shifts the conduction band (CB) below *E*_F_, which is why the electron pockets at *Λ* are visible in the ARPES spectrum. This reveals the existence of an indirect bandgap (*E*_g_) that can be quantified by taking the difference of the energy positions of the CB minimum (at the *Λ* point) and the VB maximum (at the *Γ* point) from two energy distribution curves (EDCs) of the ARPES spectrum (taken along the dashed lines in Fig. 2d). As shown in Fig. 2f, we extract a bandgap value of *E*_g_ = 120 ± 20 meV centered at *E* = −110 meV ± 20 meV. The observed band dispersion and gap value is characteristic of band inversions predicted for 1*T*′-TMD materials^8^.

**Fig. 3:**
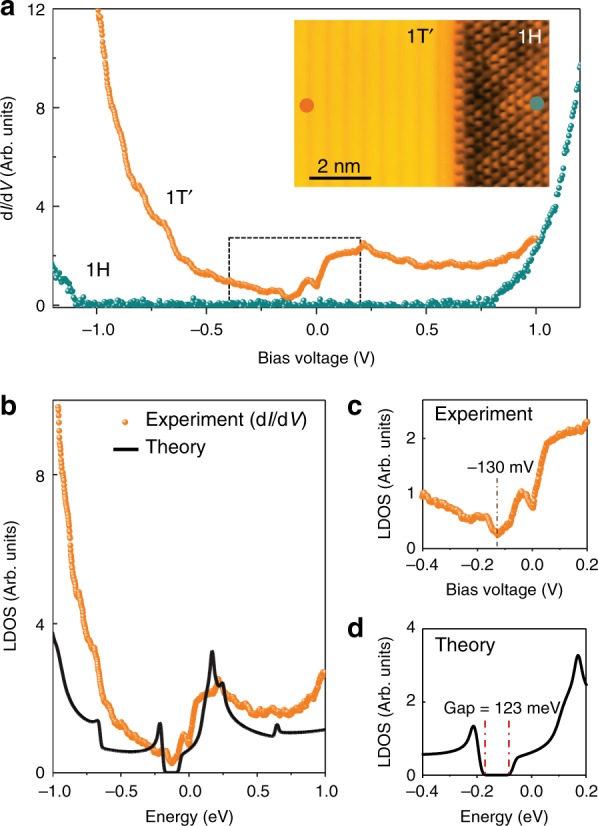
STS characterization of single-layer mixed-phase WSe_2_. **a** STS spectra obtained in the 1*T*′ (orange) and 1*H* (blue) regions of single-layer WSe_2_ (*f* = 614 Hz, *I*_t_ = 0.3 nA, *V*_rms_ = 4 meV). The inset shows an STM image of coexisting 1*T*′ and 1*H* regions with a well-ordered interface between them (*V*_s_ = +500 mV, *I*_t_ = 0.1 nA). **b** Calculated LDOS(E) of bulk single-layer 1*T*′-WSe_2_ (black curve) compared to experimental STS spectrum (orange curve). **c** Close-up view of the boxed region in **a** shows low-energy experimental STS spectrum taken for 1*T*′-WSe_2_ phase. **d** Calculated LDOS(E) for 1*T*′-WSe_2_ over the same energy range as in **c**

The local density of states (LDOS) of mixed-phase, single-layer WSe_2_ was measured via STS point spectroscopy, as seen in Fig. 3a. The 1*H* phase of monolayer WSe_2_ shows a bandgap of 1.94 eV, in good agreement with previous measurements^19^, but the 1*T*′ phase reveals a finite, asymmetric LDOS that extends across both the occupied state and unoccupied state regions. The most pronounced feature in the unoccupied state region of the 1*T*′ phase is a broad, asymmetric peak centered around + 0.24 V. The finite LDOS seen in the occupied state region of the 1*T*′ phase (−1 V < *V*_s_ < 0 V) confirms that the bands observed in ARPES at low binding energy (Fig. 2d) belong to the 1*T*′ phase since this energy range is clearly gapped out for the 1*H* phase. Also prominent in the electronic structure of the 1*T*′ phase is a gap-like feature located at *V*_s_ = −130 ± 5 mV. Figure 3c shows a close-up of this feature (the boxed region of Fig. 3a). The width of this 1*T*′ gap feature can vary depending on surface position, but it has an average FWHM = 85 mV ± 21 meV (see Supplementary Note 3 for gap statistics). A second dip feature located at *E*_F_ can be seen in the d*I*/d*V* curves taken for 1*T*′-WSe_2_. A similar zero-bias feature has also been seen in 1*T*′-WTe_2_ and has been attributed to the opening of a Coulomb gap^23^. These characteristic features are seen throughout the 1*T*′ bulk region for islands with the narrowest widths larger than ~8 nm. For 1*T*′ islands of smaller widths the zero-bias feature is replaced by a larger size-dependent energy gap that opens at *E*_F_ and dominates the electronic structure, ostensibly due to size quantization effects^24^. The bulk gap feature observed by STM spectroscopy at *V*_S_ = −130 mV is consistent with the ARPES bulk bandgap for 1*T*′-WSe_2_ when lifetime broadening effects are taken into account (Supplementary Note 3). Such broadening likely arises from a combination of electronic, vibrational, and defect-based scattering, as well as coupling to the graphene substrate^25^.

**Fig. 4:**
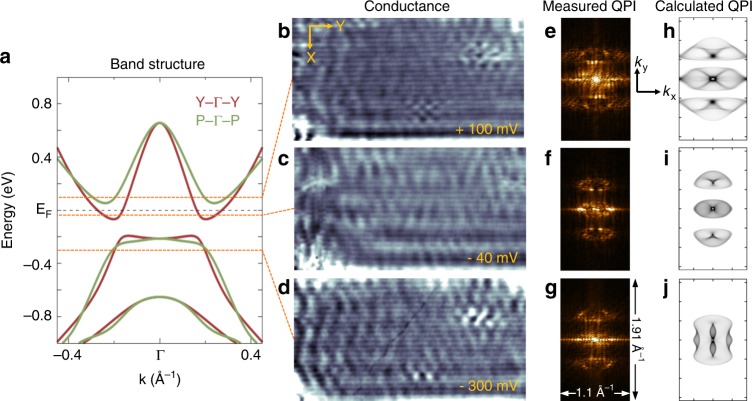
Quasiparticle interference patterns in single-layer 1*T*′-WSe_2_. **a** Calculated band structure of single-layer 1*T*′-WSe_2_ along *Γ-Y* (brown) and *Γ-P* (green) directions in the ± 1 eV range. **b**–**d** Experimental d*I*/d*V* conductance maps taken at **b** *V*_s_ = +100 mV, *I*_t_ = 0.15 nA, **c** *V*_s_ = − 40 mV, *I*_t_ = 0.15 nA, and **d** *V*_s_ = − 300 mV, *I*_t_ = 0.15 nA (14 nm × 26.4 nm, *f* = 614 Hz, *V*_rms_ = 4 meV). **e**–**g** FFTs of the conductance maps in **b**–**d**. **h–j** Calculated QPI patterns for **h** *E* =+100 meV, **i** *E* = − 40 meV, and **j** *E* = − 300 meV

In order to further understand the electronic structure of single-layer 1*T*′-WSe_2_, we also characterized its quasiparticle interference (QPI) patterns near *E*_F_ via Fourier transform (FFT) analysis of d*I*/d*V* images. Figure 4b–d show constant-bias d*I*/d*V* maps taken in the same pristine region of 1*T*′-WSe_2_ for energies within the CB (b and c) as well as in the VB (d). The QPI patterns observed in the d*I*/d*V* maps exhibit long-range oscillations with wave fronts parallel to the *x*-direction and closely spaced rows aligned parallel to the *y*-direction (i.e., the atomic rows). The corresponding FFT images of the conductance maps (Fig. 4e–g) show distinct features that reflect the band structure contours at these different energies.

**Fig. 5:**
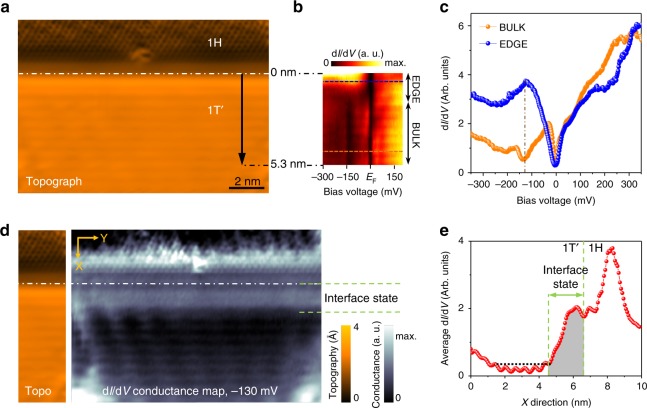
Spatial extent of atomically well-ordered 1D interface state in single-layer 1*T*′-WSe_2_. **a** STM topograph of the 1*T*′–1*H* interface (*V*_s_ = − 525 mV, *I*_t_ = 0.2 nA). Dashed line shows interface location (see text). **b** Color-coded d*I*/d*V* spectra taken along the path marked by the arrow in **a** (*f* *=* 614 Hz, *I*_t_ = 0.6 nA, *V*_rms_ = 4 meV). **c** d*I*/d*V* curves extracted from **b**. **d** Experimental d*I*/d*V* map taken in the same region as **a** for *V*_s_ = − 130 meV. Dashed line shows same interface location as in **a**. **e** Average d*I*/d*V* linescan oriented along the X direction in **d** for *V*_s_ = −130 mV

The electronic features we have described up to now for bulk single-layer 1*T*′-WSe_2_ are consistent with an inverted bandgap and the occurrence of the QSHI phase. A key feature of QSHIs, however, is the existence of helical states at the boundaries. WSe_2_ is particularly well-suited to explore the existence of such states due to the coexistence of the 1*T*′ and 1*H* phases, which leads to straight, defect-free interfaces as shown in Figs. 3a, 5a. Figure 5b shows a color-coded series of d*I*/d*V* spectra measured along the 5.3 nm-long black arrow in Fig. 5a oriented perpendicular to the 1*T*′–1*H* interface (the interface is marked by a dashed white line). The 1*T*′–1*H* interface is defined as the point where the STM topograph height reaches 50% of the height difference from the 1*H* average terrace height to the 1*T*′ average terrace height for *V*_s_ = − 0.52 V, I = 0.2 nA. This definition is also valid for other biases within the range −0.6 V < *V*_s_ < −0.1 V and I_t_ ≤ 0.5 nA (the 1*T*′ terrace is 2.9 ± 0.2 Å higher than the 1*H* terrace under these standard tunneling conditions). Figure 5b shows that the STS feature identified as the bulk bandgap at −130 meV is present in the bulk 1*T*′ material only for distances greater than 2 nm from the 1*T*′–1*H* interface.

The 1*T*′-WSe_2_ bulk gap disappears at distances closer than 2 nm from the 1*T*′–1*H* interface and a prominent peak emerges in the LDOS at the same energy that previously showed a gap. This is illustrated in Fig. 5c which shows d*I*/d*V* curves taken in the bulk region (orange curve) and in the edge region (blue curve) as indicated by the dashed lines in Fig. 5b. The emergence of this peak is consistent with the existence of a 1D topologically protected edge state as expected for a QSHI. In order to resolve the spatial extent of the interface state, we mapped the d*I*/d*V* conductance near the 1*T*′–1*H* interface with sub-nm resolution. Figure 5d shows a d*I*/d*V* map of the same region shown in Fig. 5a at the bias voltage at the center of the interface-state peak (*V*_s_ = −130 meV). This map shows bright intensity in the 1*H* phase region above the 1*T*′–1*H* interface. This is due to electronic states from the 1*T*′ phase leaking into the gapped 1*H* phase, similar to the phenomenon of metal-induced-gap-states (MIGS)^26^. Below the 1*T*′–1*H* interface in the 1*T*′ phase region a very uniform band of increased d*I*/d*V* intensity can be seen that penetrates 2 nm into the 1*T*′ bulk (marked interface state). This reveals the spatial extent of the topological interface state that resides in the bulk energy gap of single-layer 1*T*′-WSe_2_ (see Fig. 5e for average linescan profile). The penetration depth of 2 nm that we extract from this linescan is in reasonable agreement with previous predictions for topological edge states^8^. (STM spectroscopy performed at the disordered edges of 1*T*′-WSe_2_ islands also show the spectral signature of topologically protected edge states, but in this case disorder prevent any quantitative determination of edge-state width (see Supplementary Note 4).)

### Density functional theory calculations and comparison with the experiments

In order to better understand the topological behavior of this mixed-phase system, we performed ab initio calculations using density functional theory (DFT) (see Methods). The resulting relaxed structure (Fig. 1a) is consistent with previous calculations for this phase^8^ and agrees well with our STM topographic images (Fig. 1e). Figures 2e, 4a show the band structure along *Y-Γ-Y* (red) and *P-Γ-P* (green) directions over a wide energy range calculated using a hybrid functional. The results of our band structure calculations agree well with the ARPES results shown in Fig. 2 after performing a rigid shift of -130 meV to account for the n-type doping observed in our samples. The non-parabolic flattened shape of the VB near the *Γ*-point closely follows the expected band structure arising from the inversion of bands having opposite parity^27^, a prerequisite for topologically non-trivial electronic structure. The calculated band structure also shows an energy gap of 123 meV with band edges along the *ΓY* direction, in reasonable agreement with both our ARPES and STS results.

Comparison of the calculated bulk 1*T*′-WSe_2_ LDOS(E) with experimental STM d*I*/d*V* spectra shows qualitative agreement over a broad energy range as seen in Fig. 3b. The gap structure, the rise in VB LDOS as energy is decreased, and the CB peak feature near 0.2 eV are all observed. However, a quantitative comparison here would require calculating lifetime broadening effects (Supplementary Note 3) as well as energy-dependent tunneling transmission probabilities. The dip feature observed in the STS at *E*_F_ is also not captured by our calculations, likely due to its origin from, either phonon-assisted inelastic tunneling^28^ or electron-electron interactions due to the Efros-Shklovskii mechanism^23,29^. We have also simulated 1*T*′-WSe_2_ QPI patterns that take into account the band inversion and gap opening seen in Fig. 4a. Figure 4h–j show the calculated QPI patterns for energies at + 100 meV, −40 meV, and −300 meV in comparison to the experimental QPI patterns of Fig. 4e–g. Here the agreement is reasonable for features such as the multi-lobe structure along *k*_y_ and the elongation along *k*_x_, which are clearly seen for energies in the CB (Figs. 4e, h and 4f, i). In the VB (Fig. 4g, j), however, several high-intensity features in the calculated FFT are absent in the experimental data. The origin for this discrepancy may be due to either a lack of experimental resolution (due to limitations in the size of the 1*T*′ phase domains that were imaged to obtain the experimental FFTs) or to differences between the theoretical and experimental Fermi contours.

**Fig. 6:**
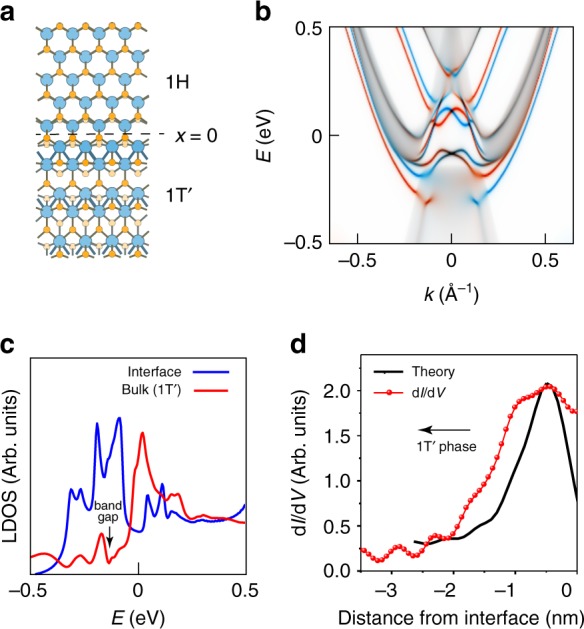
WSe_2_ 1*T*′–1*H* interface electronic structure. **a** Sketch of the structural model used to theoretically investigate the 1*T*′–1*H* interface in single-layer WSe_2_. The interface position *x* = 0 is indicated. **b** Momentum- and spin-resolved LDOS(E) at the 1*T*′–1*H* interface shows the dispersion and spin-momentum locking of the interface states (blue/red curves show different spin polarizations). **c** Energy-resolved LDOS at the 1*T*′–1*H* interface (blue curve) in single-layer WSe_2_ compared to the LDOS at a point well within the 1*T*′ bulk region (red curve). **d** Dependence of LDOS at the band gap energy on distance from the 1*T*–1*H* interface compared to experimental d*I*/d*V* linecut at *V*_s_ = −130 mV (from Fig. 5e)

The calculated electronic structure for a single-layer WSe_2_ 1*T*′–1*H* interface model structure is shown in Fig. 6. The proposed interface model (Fig. 6a) was chosen because its electronic structure best matches our experimental data. Although the experimental interface has a well-defined crystallographic orientation, it is not possible to verify its atomic structure due to limitations in experimentally resolving the chemical bonds. Our calculation of the interface electronic structure was performed using a standard DFT approach due to the large model size (see Methods). This results in a reduced band gap (29 meV) compared to the more realistic bandgap (123 meV) of the more accurate hybrid functional calculations shown in Figs. 2–4. Despite this bandgap discrepancy, it is still useful to examine the wavefunction behavior resulting from this interface structure. Figure 6b shows the calculated dispersion of topologically protected interface states running parallel to the 1*T*′–1*H* interface shown in Fig. 6a. A total of three bands span the bulk band gap. The odd number of bands is consistent with a topological origin and spin-momentum locking is clearly manifested. The pair of bands at higher energy can be attributed to Rashba-split states derived from the bulk conduction band, but the band at lower energy is topological in origin since it connects bulk valence and conduction bands. Figure 6c demonstrates how extrema in the dispersion of these interface-state bands give rise to a large LDOS intensity within the bulk bandgap (marked by the black arrow), consistent with the experimental d*I*/d*V* curve in Fig. 5c. The decay of LDOS(*E*) with distance from the 1*T*′–1*H* interface (Fig. 6d, black curve) shows that these states are localized within approximately 2 nm of the interface in the 1*T*′ domain, consistent with the experimental interface-state decay length shown in Fig. 5d, e.

In conclusion, our measurements support the results of first-principles calculations and provide evidence for the presence of the QSHI phase in single-layer 1*T*′-WSe_2_. The ability to observe 1D interface-states at atomically well-ordered boundaries between trivial and nontrivial phases allows us to extract new quantitative information on these novel states, such as their penetration depth into the 1*T*′-WSe_2_ bulk, a previously inaccessible parameter due to edge disorder. This creates new opportunities for investigating topologically non-trivial electronic phases in 2D TMDs and takes us a step closer to the integration of 2D QSH layers into more complex heterostructures that exploit topologically protected charge and spin transport.

## Methods

### Experimental details

Monolayer WSe_2_ was grown by MBE on epitaxial BLG on 6H-SiC(0001) (resistivity of *ρ* ~ 0.1  Ω  cm) at the HERS endstation of Beamline 10.0.1 (Advanced Light Source, Lawrence Berkeley National Laboratory) with a base pressure of ~3 × 10^−10^  Torr. Bilayer graphene on SiC(0001) was first prepared by following the procedure detailed in ref.^18^. To grow the TMD monolayer, pure W and Se were evaporated from an electron-beam evaporator and a standard Knudsen cell, respectively, while keeping the flux ratio of W to Se at 1:15. In order to protect the film from contamination and oxidation during transport through air to the ultrahigh vacuum (UHV) STM chamber, a Se capping layer (thickness ~10  nm) was deposited on the sample surface after growth. The Se capping layer was removed for STM experiments by annealing the sample to ~500 K in UHV for 30 min. STM and STS experiments were performed in an Omicron LTSTM operated at *T* = 4 K. The STM tip was calibrated by measuring reference spectra on the graphene substrate in order to avoid tip artifacts. STM/STS analysis and rendering was done using WSxM software^30^.

### Theoretical details

First-principles calculations were performed using DFT within the generalized gradient approximation (GGA)^31^ as implemented in the Quantum-ESPRESSO package^32^ and within the HSE03^33^ hybrid functional using the VASP package^34^. The single-particle Hamiltonian for valence and conduction states included relativistic corrections through ultrasoft pseudopotentials^35^ adapted from ref.^36^. The plane-wave basis set cutoff for wavefunctions was set to 80 Ry. Reciprocal space sampling was performed on an 11 × 18 k-point mesh in the rectangular Brillouin zone. The WSe_2_ monolayers were decoupled along the out-of-plane direction by 1.5 nm of vacuum. Prior to calculating electronic properties, the atomic coordinates and in-plane lattice constants were fully relaxed. QPI patterns were calculated via the autocorrelation function of electronic bands as implemented in WannierTools:^37^

$$f\left( {k,E} \right) = \mathop {\sum}\limits_{n,n\prime } {{\int} {\delta \left( {E_n\left( {k\prime } \right) - E} \right)} } \delta \left( {E_{n\prime }\left( {k + k\prime } \right) - E} \right)\mathrm{d}k\prime,$$

where $E_n\left( k \right)$ is the energy dispersion of the *n*^th^ Bloch band. The autocorrelation functions presented in Fig. 4h–j were calculated on a fine 1200 × 2400 k-point mesh. We find that explicitly including the matrix elements does not qualitatively change the calculated QPI patterns. The electronic structure of a 1*T*′–1*H* interface presented in Fig. 6 was calculated using the non-equilibrium Green’s function technique^38^. The Hamiltonian matrix elements were obtained in the numerical localized orbital basis set implementation^39,40^ within GGA. The atomic basis set (W7.0-s2p2d2f1 for Tungsten and Se7.0-s3p3d1 for Selenium) as well as other parameters were converged to a perfect agreement with reference GGA results of our Quantum-ESPRESSO calculations and ref. ^8^. The Hamiltonian matrix elements were obtained using models with periodic boundary conditions imposed both along and across the interface. These models contain two interfaces per supercell and measure ca. 11 nm in the direction perpendicular to the interface. Only half of the supercell containing one interface was retained for non-equilibrium Green’s function calculations, and the size of the scattering region measures approximately 6 nm.

### Data availability

The data that support the findings of this study are available from the corresponding authors upon request.

## Electronic supplementary material

Supplementary Information

Peer Review File

## References

[1] König M (2007). Quantum spin hall insulator state in HgTe quantum wells. Science.

[2] Knez I, Du RR, Sullivan G (2011). Evidence for helical edge modes in inverted InAs/GaSb quantum wells. Phys. Rev. Lett..

[3] Murakami S (2006). Quantum spin hall effect and enhanced magnetic response by spin-orbit coupling. Phys. Rev. Lett..

[4] Liu Z (2011). Stable nontrivial Z_2_ topology in ultrathin Bi(111) films: A first-principles study. Phys. Rev. Lett..

[5] Yang F (2012). Spatial and energy distribution of topological edge states in single Bi(111) bilayer. Phys. Rev. Lett..

[6] Drozdov IK (2014). One-dimensional Topological Edge States of Bismuth Bilayers. Nat. Phys..

[7] Reis F (2017). Bismuthene on a SiC substrate: A candidate for a high-temperature quantum spin Hall material. Science.

[8] Qian X, Liu J, Fu L, Li J (2014). Quantum spin Hall effect in two-dimensional transition metal dichalcogenides. Science.

[9] Tang S (2017). Quantum spin Hall state in monolayer 1*T*′-WTe_2_. Nat. Phys..

[10] Chen J (2017). Quantum effects and phase tuning in epitaxial hexagonal and monoclinic MoTe_2_ monolayers. ACS Nano.

[11] Jia ZY (2017). Direct visualization of a two-dimensional topological insulator in the single-layer 1T′-WTe_2_. Phys. Rev. B.

[12] Fei Z (2017). Edge conduction in monolayer WTe_2_. Nat. Phys..

[13] Wu S (2018). Observation of the quantum spin Hall effect up to 100 kelvin in a monolayer crystal. Science.

[14] Wang ZF (2016). Topological edge states in a high-temperature superconductor FeSe/SrTiO_3_(001) film. Nat. Mater..

[15] Pauly C (2015). Subnanometre-wide electron channels protected by topology. Nat. Phys..

[16] Sessi P (2016). Robust spin-polarized midgap states at step edges of topological crystalline insulators. Science.

[17] Wang ZF, Chen L, Liu F (2014). Tuning topological edge states of Bi(111) bilayer film by edge adsorption. Nano. Lett..

[18] Ugeda MM (2014). Giant bandgap renormalization and excitonic effects in a monolayer transition metal dichalcogenide semiconductor. Nat. Mater..

[19] Zhang Y (2016). Electronic structure, surface doping, and optical response in epitaxial WSe_2_ thin films. Nano. Lett..

[20] Cho S (2015). Phase patterning for ohmic homojunction contact in MoTe_2_. Science.

[21] Qin XR, Yang D, Frindt RF, Irwin JC (1991). Real-space imaging of single-layer MoS_2_ by scanning tunneling microscopy. Phys. Rev. B.

[22] Das PK (2016). Layer-dependent quantum cooperation of electron and hole states in the anomalous semimetal WTe_2_. Nat. Commun..

[23] Song, Y. -H. et al. Observation of Coulomb gap in the quantum spin Hall candidate single-layer 1*T*′-WTe_2_. Preprint at https://arxiv.org/abs/1711.07286 (2017). 10.1038/s41467-018-06635-x PMC6172222 30287820

[24] Han MY, Özyilmaz B, Zhang Y, Kim P (2007). Energy band-gap engineering of graphene nanoribbons. Phys. Rev. Lett..

[25] Brar VW (2010). Observation of carrier-density-dependent many-body effects in graphene via tunneling spectroscopy. Phys. Rev. Lett..

[26] Tersoff J, Hill M, Laboratories, a T. T. B. (1984). Schottky barrier heights and the continuum of gap states. Phys. Rev. Lett..

[27] Choe DH, Sung HJ, Chang KJ (2016). Understanding topological phase transition in monolayer transition metal dichalcogenides. Phys. Rev. B.

[28] Zhang Y (2008). Giant phonon-induced conductance in scanning tunnelling spectroscopy of gate-tunable graphene. Nat. Phys..

[29] Efros AL, Shklovskii BI (1975). Coulomb gap and low temperature conductivity of disordered systems. J. Phys. C. Solid State Phys..

[30] Horcas I (2007). WSXM: A software for scanning probe microscopy and a tool for nanotechnology. Rev. Sci. Instrum..

[31] Perdew JP, Ernzerhof M, Burke K (1996). Generalized gradient approximation made simple. Phys. Rev. Lett..

[32] Giannozzi P (2009). QUANTUM ESPRESSO: a modular and open-source software project for quantum simulations of materials. J. Phys. Condens. Matter.

[33] Heyd J, Scuseria GE, Ernzerhof M (2003). Hybrid functionals based on a screened Coulomb potential. J. Chem. Phys..

[34] Kresse G, Furthmüller J (1996). Efficient iterative schemes for ab initio total-energy calculations using a plane-wave basis set. Phys. Rev. B—Condens. Matter Mater. Phys..

[35] Vanderbilt D (1990). Soft self-consistent pseudopotentials in a generalized eigenvalue formalism. Phys. Rev. B.

[36] Lejaeghere K (2016). Reproducibility in density-functional theory calculations of solids. Science.

[37] Wu QS, Zhang SN, Song HF, Troyer M, Soluyanov AA (2018). WannierTools: an open-source software package for novel topological materials. Comput. Phys. Commun..

[38] Datta, S. *Quantum transport: Atom to transistor*. *Quantum Transport: Atom to Transistor* (2005).

[39] Ozaki T, Kino H (2004). Numerical atomic basis orbitals from H to Kr. Phys. Rev. B—Condens. Matter Mater. Phys..

[40] Orbital basis sets of W and Se. OpenMX Project. http://www.jaist.ac.jp/~t-ozaki/vps_pao2013/ (2013).

