# Supplementary material for: Observation of topologically protected states at crystalline phase boundaries in single-layer WSe2
Source: Nat Commun. 2018 Aug 24;9:3401. doi: 10.1038/s41467-018-05672-w (PMC6109167; doi:10.1038/s41467-018-05672-w)
Supplement: Supplementary file 1 — Supplementary Information [file 41467_2018_5672_MOESM1_ESM.pdf]

Supplementary Note 1: Morphology of single-layer 1*T*'/1*H* mixed phase WSe_2

Supplementary figure 1 shows the typical morphology of our samples grown by holding the substrate temperature at 500 K, a temperature 175 K lower than that used to grow the 1*H* phase of WSe_2 (675 K). Under these growth conditions, the surface shows a similar morphology to other MBE-grown TMDs at higher temperatures[1,2]: Large regions of single layer TMD usually decorated with small islands of bilayers. However, ~15% of the total area of WSe_2 now exhibits the 1*T*' phase, which grows both laterally to the 1*H* phase and as islands in the first and second layer. The observation of a significant fraction of the 1*T*' phase is not unexpected given the relatively small energy difference between the stable 1*H* phase and metastable 1*T*' phase (0.3 eV per unit cell)[3]. The *H* and *T*' regions as well as the straight interfaces (green arrows) are indicted in supplementary figure 1.

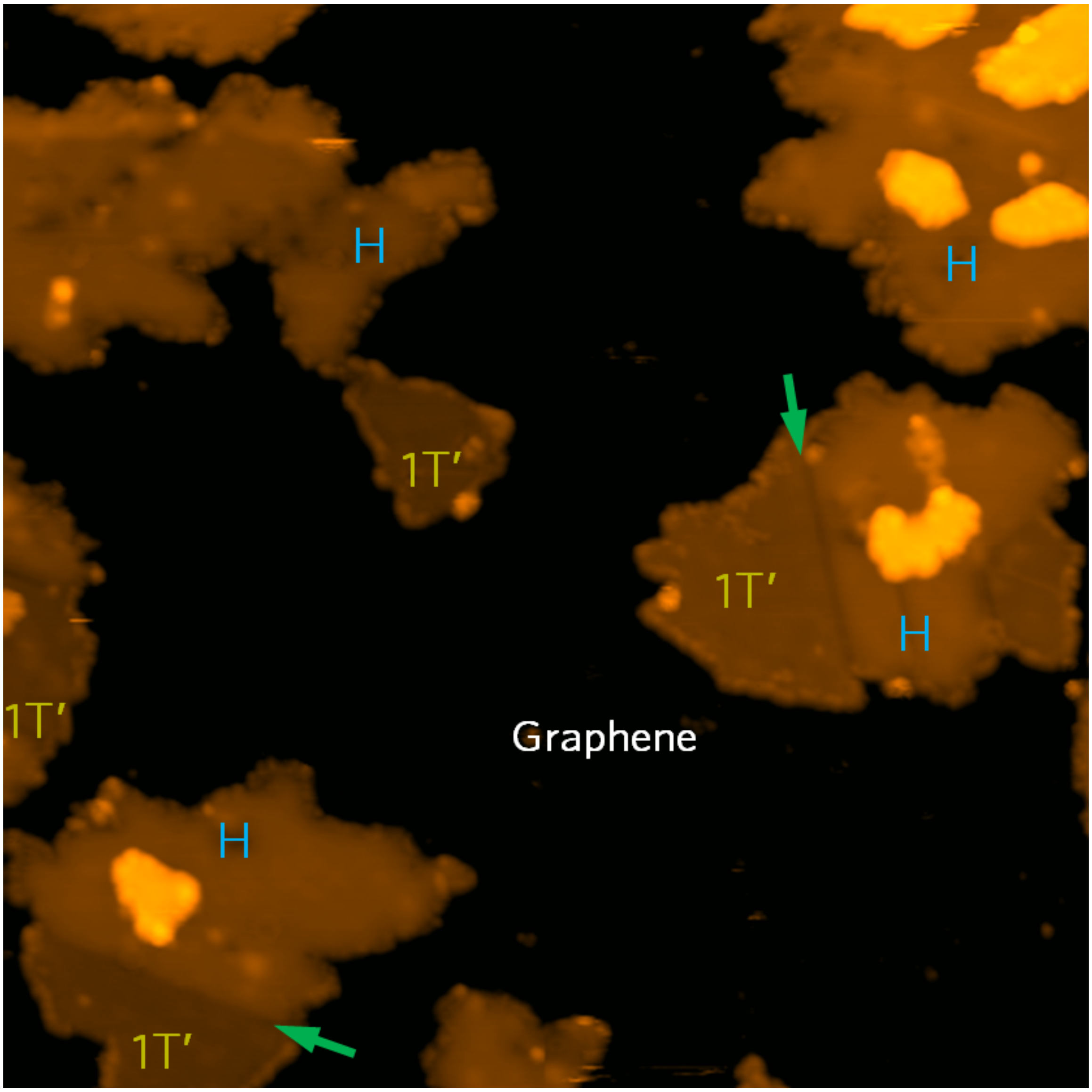

Supplementary Figure 1. STM characterization of single-layer 1*T*'/1*H* mixed phase WSe_2. STM image shows large-scale view of the WSe_2/BLG samples studied in this work. Parameters: 1000 Å x 1000 Å, V_s = + 1.8 V, I_t = 10 pA, T = 5 K.

Supplementary Note 2: ARPES Fermi surface contour of multi-domain 1*T*'-WSe_2

Since we grow the two-fold symmetric 1*T*'-WSe_2 on top of the three-fold symmetric bilayer graphene (BLG), there naturally exist three energetically equivalent domains rotated by 120° with respect to each other. Such multiple-domain structure is commonly observed, particularly when a sample and substrate have different symmetries. A well-known example of this is Bi_2Se_3 on $Bi_2Sr_2CaCu_2O_{8+\delta}$ [4,5]. Supplementary figure 2 shows how multiple domain structure in 1*T*'-WSe_2/BLG affect the observed Fermi surface (FS) through ARPES. When there exists a single domain of 1*T*'-WSe_2, the expected FS is made of two small ellipses (black) near the *Γ*-point in a rectangular surface Brillouin zone (red). The second domain in the sample, rotated by 120° compared to the first one (green), causes new FS pockets (light green) to become superimposed over the contribution from the first domain. The same holds true for the third domain (blue) and corresponding FS pockets (blue). In the extended zone scheme (bottom panel), a complex FS is obtained (black) formed by elliptical contributions from all three domains. This is well observed in our experimental data presented in figure 2.

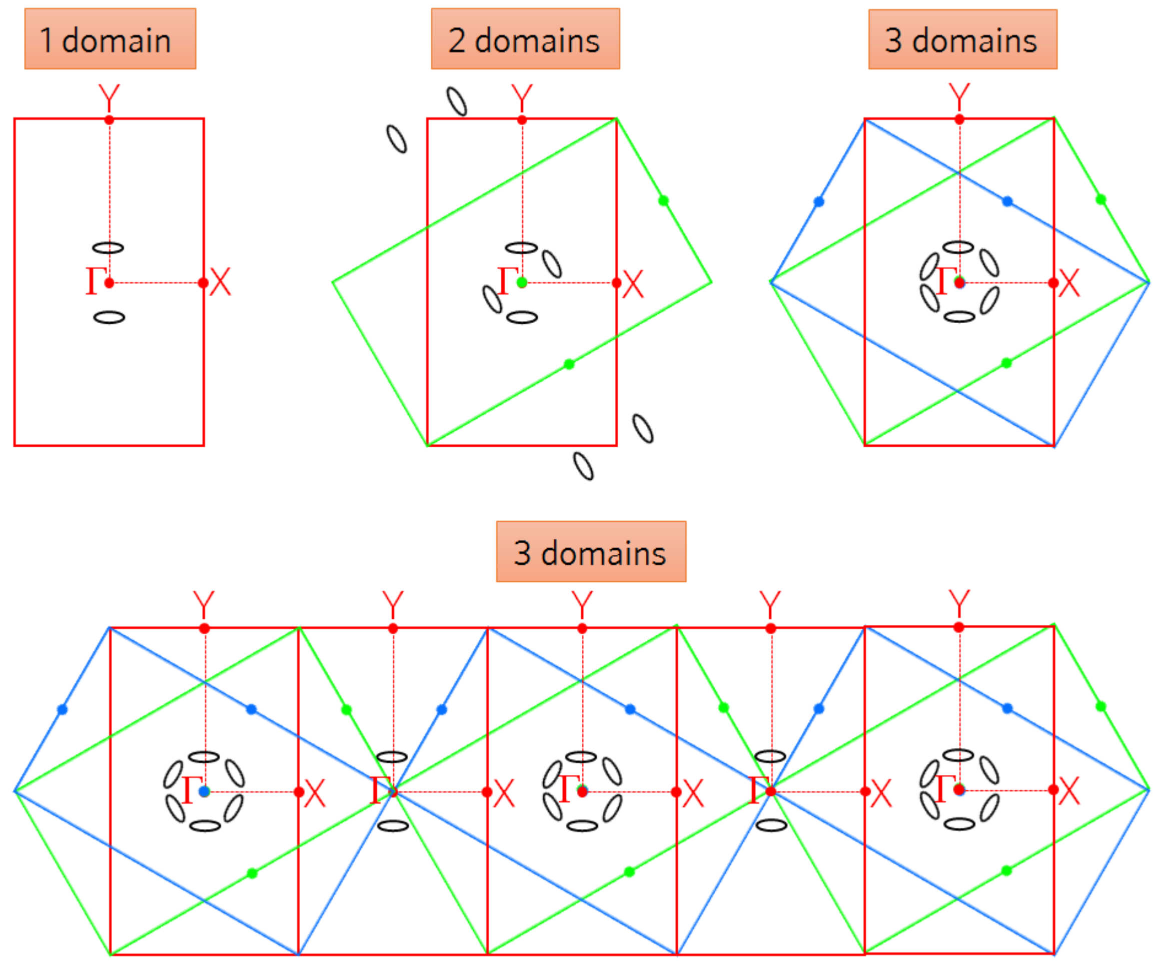

Supplementary Figure 2. Complex experimental Fermi surface topology due to the multi-domain structure of MBE grown 1*T*'-WSe_2 on bilayer graphene. The Brillouin zones corresponding to the three rotational domains are drawn in red, blue and green.

Supplementary Note 3: Single-layer 1*T*'-WSe_2 STS gap determination

The average STS gap value of 85 ± 21 mV was determined through statistical analysis of 99 d*I*/d*V* spectra collected at the surface of monolayer 1*T*'-WSe_2 for numerous different 1*T*'-WSe_2 islands and numerous different STM tips. Supplementary figure 3a shows the analysis procedure for a typical STS spectrum. We first identified the minimum of the gap (*Min*) and two peak features on both sides of the gap (P_L and P_R). The full width between the two half-height points ((Min+ P_L)/2 and (Min+ P_R)/2) is used to define the STS gap width. Analysis of 99 d*I*/d*V* spectra yields an average gap width of 85.1 mV with a standard deviation of 21 mV. A histogram of the STS gap width is shown in supplementary figure 3b.

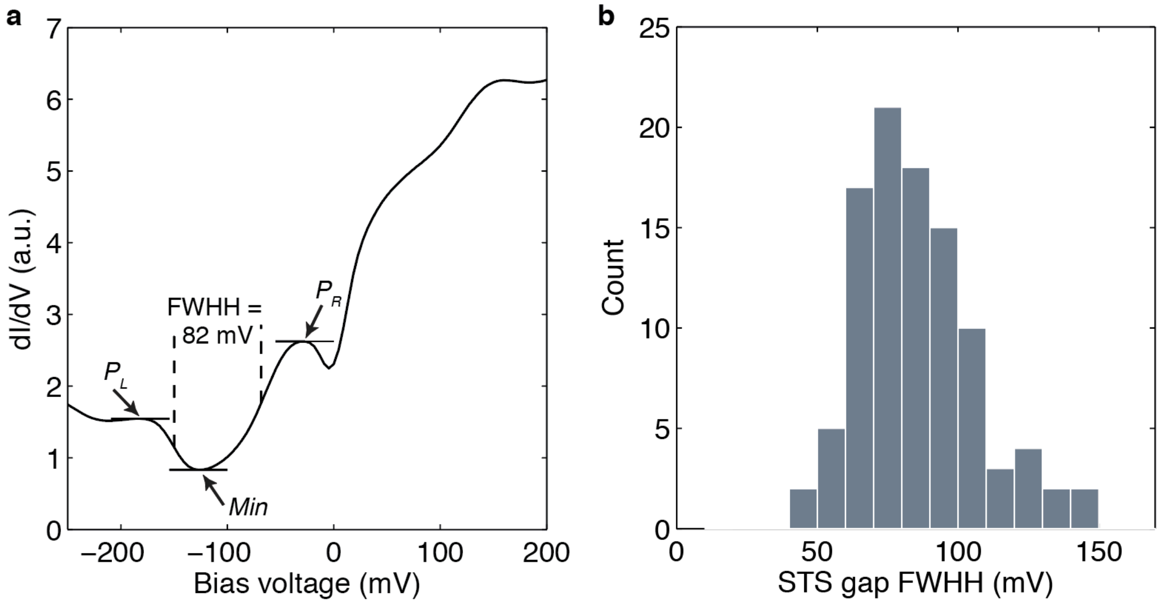

Supplementary Figure 3. STS gap determination. a, Analysis of a typical d*I*/d*V* spectrum. The minimum of the gap (*Min*) and peak features (P_L and P_R) are identified. The full width between the two half-height points ((Min+ P_L)/2 and (Min+ P_R)/2) is used to define the STS gap width. **b,** Histogram of the STS gap width.

The STS gap width defined this way (85 ± 21 meV) is smaller than the measured ARPES gap (120 ± 20 meV) and the band gap calculated using the hybrid functional DFT approach (123 meV). The observed STS spectra also finite spectral weight inside the gap rather than showing a full gap as expected from the DFT calculations. This can be explained by lifetime broadening in the STS spectra of single-layer 1*T*'-WSe_2 as shown in supplementary figure 4.

Supplementary figure 4 compares the simulated lifetime-broadened LDOS curves (supplementary figures 4b-g) to a typical experimental dI/dV spectrum (supplementary figure 2h). The lifetime-broadened LDOS curves were calculated by convoluting the LDOS spectrum (supplementary figure 3a) with a Lorentzian function having a broadening parameter Γ[6]

$$\mathrm{LDOS}(E,\Gamma) = \int dE' \frac{\Gamma}{(E-E')^2+\Gamma^2} \times \mathrm{LDOS}(E',0) \qquad (1)$$

As Γ is increased, the spectral weight inside the gap fills up. Even with a small broadening of Γ = 10 meV, the gap is no longer full. A FWHH analysis of the broadened LDOS curves shows that the effective gap size decreases with increasing Γ. When Γ is 30 meV, the overall shape of the broadened LDOS curve strongly resembles the experimental dI/dV spectrum, implying the significance of lifetime broadening in the collected STS spectra.

It is worth noting that lifetime effects should also broaden the ARPES spectra. An estimation of Γ = 30 meV corresponds to FWHH = 60 meV, which is comparable to the FWHH of the ARPES EDC at gap edges in figure 2f. However, extracting the exact

quasiparticle lifetime from the ARPES spectra requires a detailed understanding of all sources of inhomogeneous broadening (e.g. defects), which we presently do not have.

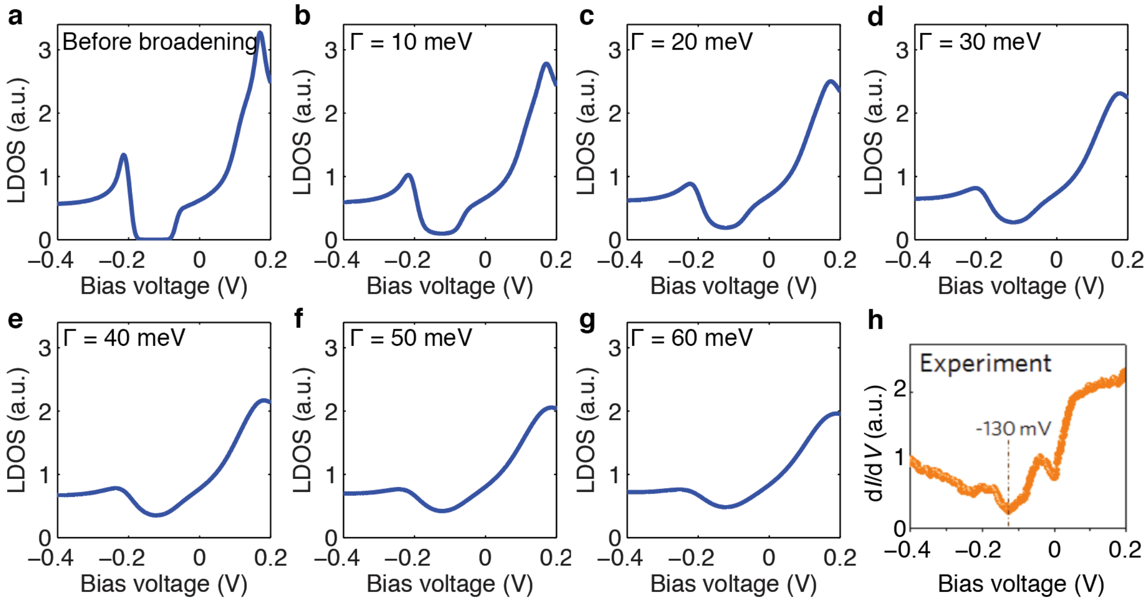

Supplementary Figure 4. Comparison between lifetime-broadened LDOS curves and an experimental STS spectrum. a, Theoretical LDOS spectra before broadening. **b-g**, Broadened LDOS curves with different broadening parameters Γ. **h,** Experimental d*I*/d*V* spectrum.

Supplementary Note 4: Observation of topologically protected edge states at irregular edges

The topological protection of boundary states in a QSHI phase implies that they should survive regardless of the structural features of the boundary, including different levels of interface roughness and defects, so long as time-reversal symmetry is preserved. In order to test this prediction in single-layer 1*T*'-WSe_2 we performed STS measurements of disordered edges of 1*T*'-WSe_2 islands as shown in supplementary figure 5a. Supplementary figure 5b shows a color-coded plot of a series of d*I*/d*V* curves taken along the black arrow in supplementary figure 5a, which demonstrate a transition in electronic behavior from bulk to edge. Similar to the 1*T*'/1*H* interface, the gap feature at –130 meV abruptly disappears in a narrow strip near the edge and a peak emerges in the LDOS at the bulk gap energy. Here, however, the width of the edge state cannot be accurately measured due to disorder in the structure of the 1*T*'-vacuum interface. The fact that we observe the edge states regardless of edge structural details provides further evidence that we are observing a topologically-protected edge state rather than a trivial one.

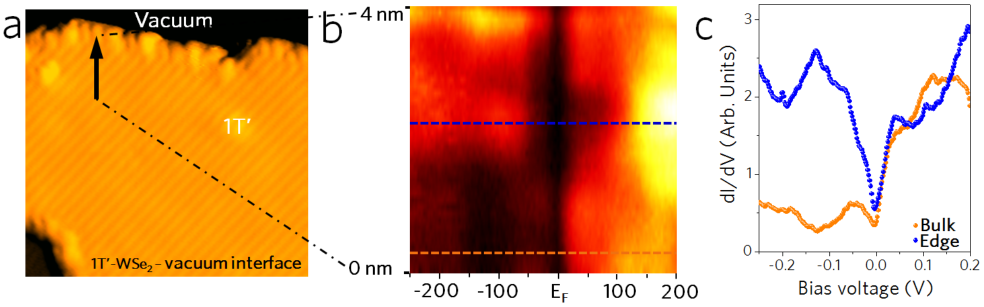

Supplementary Figure 5. Helical edge-state at disordered edges. a, STM image of a 1*T*'-WSe_2 monolayer with irregular edges (17 nm x 16 nm, V_s = + 1000 mV, I_t = 0.01 nA). **b**, Color-coded d*I*/d*V* spectra taken along the path marked by the black arrow in **a** (f = 614 Hz, I_t = 0.1 nA, V_{rms} = 4 meV). **c**, d*I*/d*V* curves extracted from **b**.

References

1. Ugeda, M. M. *et al.* Giant bandgap renormalization and excitonic effects in a monolayer transition metal dichalcogenide semiconductor. *Nat. Mater.* **13,** 1091–1095 (2014).
2. Zhang, Y. *et al.* Electronic structure, surface doping, and optical response in epitaxial WSe_2 thin films. *Nano Lett.* **16,** 2485–2491 (2016).
3. Qian, X., Liu, J., Fu, L. & Li, J. Quantum spin Hall effect in two-dimensional transition metal dichalcogenides. *Science* **346,** 1344–1347 (2014).
4. Wang, E. *et al.* Fully gapped topological surface states in Bi_2Se_3 films induced by a d-wave high-temperature superconductor. *Nat. Phys.* **9,** 621–625 (2013).
5. Ugeda, M. M. *et al.* Characterization of collective ground states in single-layer $NbSe_2$. *Nat. Phys.* **12,** 92–97 (2016).
6. Brar, V. W. *et al.* Observation of Carrier-Density-Dependent Many-Body Effects in Graphene via Tunneling Spectroscopy. *Phys. Rev. Lett.* **104,** 36805 (2010).
